# Comparison of a Commercial ELISA with the Modified Agglutination Test for Detection of *Toxoplasma gondii* Antibodies in Sera of Naturally Infected Dogs and Cats

**Published:** 2012

**Authors:** CH Zhu, LL Cui, LS Zhang

**Affiliations:** 1 School of public health, Sichuan University, Chengdu, 610041, China; 2 College of Veterinary Medicine, China Agricultural University, Beijing, 100193, China

**Keywords:** *Toxoplasma gondii*, Dogs, Cats, Modified agglutination test, ELISA

## Abstract

**Background:**

*Toxoplasma gondii* can infect all warm-blooded animals. Modified agglutination test (MAT) and ELISA are widely used for the detection of *T. gondii* antibodies. However, there is little information on their acceptability for detecting antibodies in companion animals.

**Methods:**

This study compared ELISA and MAT for their ability to detect *T. gondii* infection in naturally infected dogs and cats. Blood samples were collected from dogs and cats in different areas of Beijing, China and analyzed by ELISA and MAT. The χ^2^ test and κ analysis were used to evaluate their efficiency and agreement.

**Results:**

For dogs, the seroprevalence of *T. gondii* antibodies detected by ELISA was 34.7%, which was significantly higher than that detected by MAT (*P*<0.05). There was no significant difference between ELISA and MAT for detecting *T. gondii* antibodies in cats. Good agreements between MAT and ELISA were seen in both dogs and cats; however, inconsistent results were demonstrated by κ analysis and in MAT titer assay.

**Conclusion:**

Serum-based ELISA may be more satisfactory for screening test of *T. gondii* infection in dogs, whereas both methods could be acceptable in cats.

## Introduction

Toxoplasmosis is caused by *Toxoplasma gondii* and is a disease with a major public health impact. Contact and interaction between domestic animals and humans may lead to an increased risk of transmission of *T. gondii* (1). *Toxoplasma gondii* is a coccidia and has evolved in cats (definitive host), where sexual multiplication occurs (2). This results in dissemination of many oocysts into the environment, where they can infect all types of warm-blooded animals (wildlife, companion animals, domestic livestock), including humans (intermediate host).

Intermediate hosts can be infected by ingesting food or water contaminated with oocysts, eating undercooked meat with tissue cysts, or by transplacental infection with tachyzoites (2). Prevention and control of the prevalence of *T. gondii* and effective prevention of toxoplasmosis in animals and humans require good diagnostic or testing methods that are appropriate for the specific species. There are many testing methods for *T. gondii* detection in animals and humans (3, 4), however, serological tests have some distinct advantages that have been described elsewhere (5–7). Among the serological tests, modified agglutination test (MAT) and ELISA have been widely used both in clinical and epidemiological surveys for screening of *T. gondii* infection. However, evaluation of their diagnostic capability for detecting *T. gondii* infection in animals has focused solely on some domestic animals (4, 8–10), and few studies have investigated MAT and ELISA for detection of *T. gondii* antibodies in naturally infected companion animals.

In the present study, the efficiency of a commercial SPA-ELISA and MAT for detecting *T. gondii* antibodies in sera of naturally infected pet dogs and cats was compared, and their intrinsic agreement was also evaluated through analysis of κ statistics. Considering the lack of the information on the sensitivity and specificity of MAT and ELISA methods in dogs and cats, an MAT titer assay was also performed to evaluate the potential agreement between the methods.

## Materials and Methods

Blood samples from 1876 dogs and 762 cats were collected from different localities of Beijing, China, including the animal hospital affiliated to China Agricultural University, and animal welfare shelters of Haidian district, from May 2010 to April 2011. Blood samples were obtained from the jugular or hind limb veins, and then centrifuged at 3500 rpm for 10 min for serum separation after coagulation. Separated sera were labeled and stored at –20°C for the following test. The serum samples were diluted 1:25 for the comparative analysis (MAT) and diluted in twofold serial dilutions from 1:10 to 1:640 for the MAT titer assays.

For comparison of the two testing methods, 121 serum samples from dogs and 45 from cats were randomly selected, and *T. gondii* antibodies were detected using ELISA and MAT. For the MAT titers assay, 417 serum samples from dogs and 263 from cats were randomly selected and detected first by ELISA, and then positive samples were tested by MAT at different titers.

Serum IgG antibodies to *T. gondii* were detected using a commercial ELISA kit (Toxo SPA-ELISA kit, Haitai Bio., Shenzhen, China) according to the manufacturer's instructions. Whole *T. gondii* tachyzoites (as antigen) were coated on a 96-well plate. After incubation of the diluted serum sample (1:100) in the test well and subsequent washing, a horseradish-peroxidase-conjugated staphylococcal protein A was added as second antibody, which combined with the Fc fragment of IgG and had no species specificity. The plate was washed again and the chromogenic enzyme substrate was added. OD_450_ was read using iMark™ Microplate Absorbance Reader (Bio-Rad Laboratories, Hercules, CA, USA)

MAT was performed according to the standard procedures (11, 12). MAT was performed with a suspension of *Toxoplasma* tachyzoites fixed with formalin, serum samples diluted in PBS (pH 7.2), positive and negative control sera, antigen-diluting buffer containing bovine serum albumin (BSA), 2-mercaptoethanol (to deplete the serum of non-specific IgM), and Evans blue dye solution. MAT titers of 1:25 or higher were considered as positive. Positive and negative controls were incorporated in each test and tested at the same dilutions of serum samples. A negative result was when the base of the “U” bottom 96-well microtiter plates contained a blue pellet; conversely, a clear bottom indicated a positive result.

All statistical analyses were conducted using SPSS version 15.0. A χ^2^ test was used to analyze the difference in efficiency and *P*<0.05 was considered significant. κ analysis was used to evaluate the agreement between MAT and ELISA for the detection of *T. gondii*-specific antibodies.

## Results

Paired serum samples were randomly selected and tested using the two methods. Of the 121 samples randomly selected from dogs, 42 (34.7%) were positive by ELISA and 28 (23.1%) were positive by MAT (Table 1). The χ^2^ test suggested that the positive rate of antibodies to *T. gondii* in naturally infected dogs differed significantly between the two methods (χ^2^=3.940, *P*<0.05). However, κ analysis demonstrated that the two methods had a high degree of agreement in detecting *T. gondii* infection in dogs (κ=0.644).

**Table 1:** The inter-agreement between MAT and ELISA in the course of detecting *T. gondii* antibodies in pet dogs

MAT	ELISA		Total(Rate)
	+	－	
+	26	2	28 (23.1%)
－	16	77	93 (76.9%)
Total (Rate)	42 (34.7%)	79 (65.3%)	121
Weighted Kappa (κ)			0.644
Standard error			0.074
95% CI			0.499 to 0.789

χ^2^=3.940, *P*<0.05.

Among the 45 serum samples from cats, 16 (35.5%) were positive by ELISA and 11(24.4%) by MAT (Table 2). The χ^2^ test suggested that there was no significant difference between the positive rates of *T. gondii* antibodies detected by the two methods (χ^2^=1.323). The corresponding κ value decreased to 0.531, although it still demonstrated a moderate agreement between the two methods in detecting *T. gondii* infection in cats.

**Table 2:** The inter-agreement between MAT and ELISA in the course of detecting *T. gondii* antibodies in pet cats

MAT	ELISA		Total(Rate)
	+	－	
+	9	2	11 (24.4%)
－	7	27	34 (75.6%)
Total (Rate)	16 (35.6%)	29 (64.4%)	45
Weighted Kappa (κ)			0.531
Standard error			0.133
95% CI			0.270 to 0.791

χ^2^=1.323, *P*>0.05.

Ninety-two ELISA-positive samples from 417 naturally infected dogs, and 53 from 263 cats were further tested for MAT titer (Table 3).

**Table 3:** MAT titers for 92 ELISA-positive dogs and 53 ELISA-positive cats

Samples	MAT(titer)							
	0	1:10	1:20	1:40	1:80	1:160	1:320	1:640
ELISA-positive Dogs (n=92)	19	22	17	15	5	4	8	2
	20.7%	23.9%	18.5%	16.3%	5.4%	4.3%	8.7%	2.2%
ELISA-positive Cats (n=53)	5	5	3	3	2	4	10	21
	9.4%	9.4%	5.7%	5.7%	3.8%	7.5%	18.9%	39.6%

**Fig. 1:**
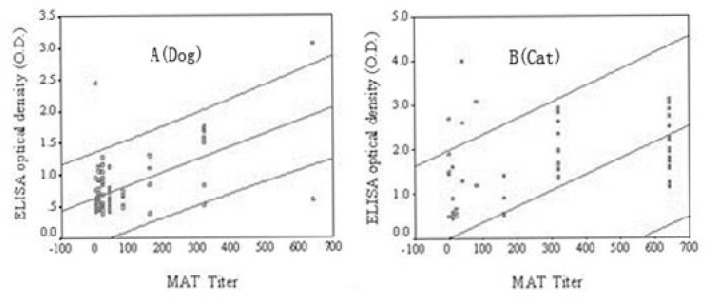
Relationship between MAT titer and ELISA OD for 92 dogs and 53 cats. Data shown for individual dogs and cats were fitted to a linear regression. A and B show the agreement analysis through straight line regression between MAT titer and ELISA OD detected in dogs and cats, respectively. The middle line is the regression line, and the whole area between the outer lines is the prediction interval

The relationship between MAT titer and ELISA OD values in dogs and cats is shown in Fig. 1, which showed a positive correlation between MAT titer and ELISA OD. Fig. 1 shows that there was a better goodness-of-fit of regression between ELISA OD value and MAT titer in dogs than in cats (95.6% versus 92.4%).

Such a result further demonstrated that there was a better agreement between ELISA and MAT in detecting of *T. gondii* antibodies in dogs. Table 3 indicates that cats had a higher titer than dogs. Twenty-one (39.6%) of the 53 serum

samples from cats reached a titer of 1:640, while only two (2.2%) of the 92 serum samples from dogs reached this titer. If we use a titer of 1:10 as a cut-off value for MAT, among the ELISA-positive serum samples, 73 (79.3%) from dogs and 44 (83.0%) from cats were seropositive for *T. gondii* antibodies. However, if we use 1:20 as a positive cut-off value for MAT, the corresponding seropositivity rate decreased to 55.4% for dogs and 81.2% for cats.

## Discussion

Diagnosis of toxoplasmosis by demonstration of *T. gondii* in tissue is too difficult. Cat/ mouse-based bioassays are not suitable for use in the field or for rapid detection, due to the length of time required to obtain a result, or their applicability and reliability, and other reasons that have been described elsewhere (5–7). Serological tests are still considered useful for epidemiological surveys and for estimating infection rate of *T. gondii*, which helps to prevent and control its transmission through improving management and reducing potential human exposure, because there is still no suitable human vaccine (7).

There are many references to serological tests for *T. gondii* in the literature, including indirect hemagglutination test (IHAT), indirect fluorescent antibody test (IFAT), latex agglutination test (LAT), ELISA and MAT, which have been widely used to detect *T. gondii* antibodies in sera of animals and humans (4, 7, 13). The sensitivity and specificity of the various tests differ according to sampling methods and reference populations, and even when the same tests are used in different species (13–15). Several problems associated with the available methods for serodiagnosis of *T. gondii* infection have been an impetus to search for alternative methods.

ELISA and MAT are the most widely used methods for *T. gondii* detection worldwide (3). However, most of the data on MAT and ELISA have been obtained from parasites isolated from naturally and experimentally infected domestic animals (3, 9), with few tests based on companion animals. When a gold standard is not available, at least two different tests should be performed (16); however, it is difficult and impractical to conduct at least two diagnostic tests in practice, especially for epidemiological surveys or high-throughput screening. In the present study, we compared an available commercial ELISA with MAT for detection of *T. gondii* antibodies in sera of naturally infected dogs and cats.

In this study, we found a significant difference between the two tests for detection of *T. gondii* antibodies in sera from dogs. A high seroprevalence of *T. gondii* antibodies in dogs (34.7%) was detected using ELISA, but MAT showed that only 23.1% of the sera were seropositive at a cut-off titer of 1:25, which was higher than that detected previously using MAT (10.81%) in Lanzhou, China (17). This result was consistent with a previous study in domestic pigs (9), which also has suggested that ELISA may be a good tool for epidemiological studies of *T. gondii* infection in pigs. However, the opposite conclusion was reached in another study in naturally infected sheep (10). There was no significant difference between the corresponding seropositivity rates in pet cats detected by ELISA and MAT. This suggests that both ELISA and MAT are suitable for testing the seroprevalence of *T. gondii* antibodies in cats. However, κ analysis demonstrated that there was a better agreement between ELISA and MAT for the detection of *T. gondii* antibodies in dogs than in cats. Taken together, our results suggest that, for accurate diagnosis of *T. gondii* in pet animals, ELISA should be recommended for primary screening, but the screening result may need to be adjusted by MAT.

Reliability and agreement are basic quality requirements of diagnostic tests; however, the way in which reliability and agreement studies are conducted, reported and interpreted seems not to be straightforward (18). Here, the concern is with how well these tests agree, and not with their relationship with the gold standard or true diagnosis. Reliability here is the extent to which the results of different test methods agree, and not merely the extent to which the results are associated or correlated. In such a context, an agreement test should be used.

The κ test remains the most commonly used measure (19, 20), mainly because it provides a measure of agreement beyond that which would be expected by chance, as estimated by the observed data. In the present study, κ analysis was used to determine the potential agreement between ELISA and MAT in detecting *T. gondii* antibodies in pet dogs and cats. The result showed that there was a good agreement between the two test methods in both species, although there was a slightly better agreement in dogs (0.644 versus 0.531).

Due to the lack of references to the sensitivity and specificity of the two methods for detecting *T. gondii* antibodies in sera from pet dogs and cats, in this study, we used the MAT titers to analyze the seropositivity rate of *T. gondii* antibodies, which had been detected by ELISA. We found that there was a higher MAT titer in the sera from ELISA-positive cats; in contrast, the number of ELISA-positive dogs with high MAT titer was small. In this regard, such results may display a higher degree of agreement between the two methods in cats than in dogs, which was not consistent with the κ analysis. Notably, agreement studies should not be confused with studies of accuracy in which measures of sensitivity and specificity are commonplace for comparisons when a reference or gold standard exists.

In the present study, the differences in agreement between the two methods in dogs and cats might be attributed to the different sensitivity and specificity between ELISA and MAT in detecting *T. gondii* antibodies, and differences in immune status, age, living conditions, or species susceptibility. An apparent difference in the agreement obtained through κ analysis and our MAT titers assay was also noted. This could have resulted from the intrinsic difference between the two methods, such as there being no default paired samples in MAT titer analysis. It could also have been due to the difference in immune status or species susceptibility, for example, a lower MAT titer was found in dogs than in cats, which has also been found in other studies (17, 21).

The advantages of ELISA over other diagnostic methods are that it can be semi-automated, the procedures can easily be conducted, and the results can be read objectively without the need for experienced personnel. This means that it can be used for screening many samples rapidly and is ideal for epidemiological surveys worldwide. For MAT, although it does not require species-specific conjugates, the length of time needed and the subjective nature of results interpretation may render it impractical for widespread application in clinical diagnosis or epidemiological surveys.

As far as we are aware, the present study is the first to compare MAT and ELISA for detection of *T. gondii* antibodies in serum samples from naturally infected cats and dogs from Beijing, China. We demonstrated that ELISA performed better than MAT for detecting *T. gondii* serum antibodies in dogs, whereas there was no significant difference between the seropositivity rates for *T. gondii* antibodies detected by the two methods in cats. At the same time, κ analysis showed that the two methods could be considered to have good agreement for detection of *T. gondii* antibodies in dogs and cats, although a slightly better agreement was found in dogs. We think that the variation between the results of the two serological tests might have been due to differences in sensitivity and specificity, and to differences between the animal species. We assumed that the reliability of SPA-ELISA and MAT was similar between the two groups of animals; however, we did not select them according to their biological factors such as age, sex, living conditions, physiological status, stage of disease, and species susceptibility, which might also have influenced the sensitivity and specificity of the methods.

In conclusion, our results demonstrated good agreement between ELISA and MAT for the detection of *T. gondii* antibodies in both dogs and cats. However, a serum-based ELISA would be more satisfactory for epidemiological survey of toxoplasmosis in dogs, whereas for cats, both methods could be used. For accurate diagnosis, the screening result may need to be adjusted by MAT.
